# Impfen: Prävention von Infektionskrankheiten und ihren Folgen

**DOI:** 10.1007/s00103-025-04033-5

**Published:** 2025-03-27

**Authors:** Maren Mylius, Joseph Kuhn

**Affiliations:** 1 Abteilung 6 „Öffentliche Gesundheit“, Referat 633 „Impfungen, STIKO“, Bundesministerium für Gesundheit, Mauerstr. 29, 10117 Berlin, Deutschland; 2 Pettenkofer School of Public Health München, c/o Bayerisches Landesamt für Gesundheit und Lebensmittelsicherheit, Veterinärstr. 2, 85764 Oberschleißheim, Deutschland

„Kinderlähmung ist grausam, Schluckimpfung ist süß“, der Slogan der Kampagne für die Schluckimpfung gegen Polio in den 1960er-Jahren in der Bundesrepublik Deutschland ist bei vielen Menschen immer noch präsent. Der große Erfolg der Impfung zeigte sich im rasanten Rückgang der Polio-Fallzahlen in den Folgejahren. Im Jahr 1990 wurde der letzte Fall von in Deutschland erworbener Polio durch das Wildvirus gemeldet. Die Einführung von Impfungen führte auch bei anderen Erkrankungen zu einem drastischen Rückgang von Erkrankungsfällen. Zu den größten Erfolgen gehört die Eradikation der Pocken, die von der Weltgesundheitsorganisation (WHO) 1980 erklärt wurde. Sozioökonomische und systemische Veränderungen, wie beispielsweise die Verbesserung der materiellen Lebensbedingungen und der Bau von Kanalisationsanlagen, aber auch die Einführung von Antibiotika, haben zusammen mit Impfungen die allgegenwärtigen Seuchen von damals in vielen Ländern zurückgedrängt. Mit der „epidemiologischen Transition“ zeigte sich eine grundlegende Veränderung der häufigsten Todesursachen in vielen Gesellschaften.

Für die Reduktion von impfpräventablen Erkrankungen bleibt die breite Inanspruchnahme von Impfungen von hoher Relevanz. Doch die aktuell vorliegenden Auswertungen durch das Robert Koch-Institut zeigen teils erhebliche Lücken in den Impfquoten in Deutschland. Routineimpfungen für Säuglinge und Kleinkinder werden oftmals später durchgeführt als empfohlen oder Kinder und Jugendliche werden gegen einzelne Erreger überhaupt nicht geimpft. Bei Erwachsenen bestehen oft noch erheblich größere Impflücken. Die Impfquoten weisen außerdem große regionale Unterschiede auf, sowohl zwischen den Bundesländern als auch kleinräumig innerhalb der Länder.

Die Grundlage für die breite Durchführung von Impfungen in Deutschland ist die sorgfältige evidenzbasierte Abwägung von Nutzen und Risiken der Anwendung. Die Ständige Impfkommission (STIKO) beim Robert Koch-Institut entwickelt als unabhängiges Gremium von Expertinnen und Experten Impfempfehlungen für die Bevölkerung. Nach einer transparenten, standardisierten Vorgehensweise wird die verfügbare Evidenz analysiert, um das individuelle Nutzen-Risiko-Verhältnis sowie die Effekte auf Bevölkerungsebene zu bewerten. Für die von der STIKO empfohlenen Impfungen fällt die Abwägung – immer gemessen am aktuellen Stand des Wissens – eindeutig zugunsten des Nutzens der Impfung aus.

Durch Impfungen werden Infektionen und die damit verbundenen Erkrankungen sowie Folgeleiden reduziert. Mit den Impfungen gegen humane Papillomviren (HPV) und Hepatitis B kann beispielsweise das Auftreten bestimmter Krebserkrankungen vermieden bzw. das Risiko erheblich verringert werden. Viele Impfungen vermitteln nicht nur einen Individualschutz, sondern haben darüber hinaus einen Gemeinschaftseffekt, im Idealfall eine Herdenimmunität. Dadurch werden auch Menschen geschützt, die nicht geimpft werden können oder trotz Impfung keinen ausreichenden Immunschutz aufbauen. Immunsupprimierte Patientinnen und Patienten gehören dazu, eine Gruppe, die durch die Entwicklung von neuen Therapien in den letzten Jahrzehnten deutlich zugenommen hat. Durch hohe Impfquoten kann der Einsatz von Antibiotika gesenkt und der Entwicklung antimikrobieller Resistenzen entgegengewirkt werden. Bei einigen Erregern, bei denen der Mensch das einzige Infektionsreservoir ist (z. B. Masern, Polio), könnte sogar eine weltweite Eradikation erzielt werden, wie es bei den Pocken gelungen ist. Impfungen haben zudem auch potenziell positive indirekte Effekte, wie beispielsweise die Entlastung von Gesundheitseinrichtungen und Kosteneinsparungen im Gesundheitswesen.

Durch die gesamtgesellschaftlichen Effekte waren Impfungen in den letzten Jahrzehnten nicht nur eine Frage der unmittelbaren individuellen Entscheidung, sondern immer wieder Gegenstand gesellschaftlicher Auseinandersetzung. Für die Pockenimpfung gab es in Deutschland lange eine Impfpflicht, die beispielsweise in Bayern und Baden bis Anfang des 19. Jahrhunderts zurückreicht. Die Pflicht zur Pocken-Erstimpfung ist in der Bundesrepublik 1976 und in der DDR 1982 aufgehoben worden. Das Spannungsfeld zwischen individueller Entscheidungsfreiheit, gesellschaftlichem Nutzen und regulativen Maßnahmen ist weiterhin hochaktuell – sowohl auf nationaler als auch auf internationaler Ebene.

In Deutschland gilt laut dem im Jahr 2020 in Kraft getretenen Masernschutzgesetz die einrichtungsbezogene Nachweispflicht für eine Masernimmunität. Mit der COVID-19-Pandemie hat sich zuletzt der Diskurs zu Impfungen, ihrem Nutzen, ihren Risiken, aber auch das Verhältnis von der Freiheit des Einzelnen zum Schutz der Gemeinschaft erneut entzündet und Impfdebatten nachdrücklich geprägt.

Wie bei jeder medizinischen Intervention können auch bei Impfungen Nebenwirkungen auftreten. Der Verdacht einer Nebenwirkung, einer über das übliche Maß einer Impfreaktion hinausgehenden gesundheitlichen Schädigung, ist nach dem Infektionsschutzgesetz meldepflichtig. Das Paul-Ehrlich-Institut (PEI) validiert und bewertet die gemeldeten Verdachtsfälle. Die Bedeutung einer möglichst guten Erfassung der Effekte eines Impfstoffs und der möglichen Nebenwirkungen ist in der COVID-19-Pandemie besonders deutlich geworden – davon hängt nicht nur die Möglichkeit von Anpassungen der Nutzenbewertung ab, sondern in erheblichem Maße auch das Vertrauen der Bevölkerung in die Wirksamkeit und Sicherheit von Impfungen.

Verschiedene Studien zeigen einen Zusammenhang zwischen einer niedrigen Inanspruchnahme von Impfungen und einem Informationsdefizit in der Bevölkerung, obwohl fundierte Informationen beispielsweise online über das Bundesinstitut für öffentliche Gesundheit (ehemals Bundeszentrale für gesundheitliche Aufklärung) verfügbar sind. Gleichzeitig kursiert zu diesem Themenfeld eine Vielzahl an Fehl- und Desinformationen. Vor allem die Aufklärung durch Ärztinnen und Ärzten sowie Hebammen ist wichtig für die Entscheidung zur Impfung. Sie werden als Vertrauenspersonen bei der Impfberatung wahrgenommen. Doch wie sollte eine evidenzbasierte Aufklärung stattfinden, wie eine informierte Entscheidung herbeigeführt werden? Wie können verhaltensbezogene und kulturelle Erkenntnisse („behavioural and cultural insights“) in Maßnahmen und Kommunikation zum Impfen einfließen, um Impfhemmnisse abzubauen sowie eine informierte Entscheidung zu unterstützen?

Aufsuchende Informationsangebote und ein breites Impfangebot, wie beispielsweise Schulimpfprogramme, bergen ein großes Potenzial, die Inanspruchnahme von Impfungen zu befördern. Dem Öffentlichen Gesundheitsdienst kommt hierbei eine wichtige Funktion zu, die Bevölkerung zielgruppenspezifisch zu informieren und dabei bestehende Impflücken zu berücksichtigen. Die Gesundheitsämter können darüber hinaus eine wichtige subsidiäre Aufgabe durch spezifische Impfangebote einnehmen. Welche Erfahrungen wurden in Deutschland in der Praxis gemacht und welche Hürden bestehen weiterhin?

Erinnerungssysteme für anstehende Impfungen und das Einhalten von Terminen zu medizinischen (Vorsorge‑)Untersuchungen sowie ein digitales Terminmanagement können Impfquoten erhöhen. Die Digitalisierung bietet dabei neue Chancen und muss in diesem Bereich effizient eingesetzt werden.

Erkenntnisse zu Barrieren in der Inanspruchnahme, fördernde Faktoren, Auswirkungen auf Gesellschaften und zu ökonomischen Aspekten, aber auch das Wissen zu Impfstoffen, ihrem Nutzen, ihren Risiken sind nicht statisch, sie entwickeln sich fortwährend weiter und ihre Reflexion ist unabdingbar.

Einigen der oben aufgeworfenen Fragen widmen sich die Beiträge im vorliegenden Themenheft, sie sollen einen Beitrag zur Diskussion leisten – wenngleich aufgrund der Breite des Themas in diesem Heft nur eine Auswahl an Fragestellungen aufgegriffen werden konnten –, es wird sicher eine „Fortsetzung“ geben.

Dieses Heft ist Teil einer Reihe von Heften des Bundesgesundheitsblatts zum Impfen und schließt sich an die Hefte 4/2019 und – speziell zu COVID-19 – 12/2022 an. Dabei soll der Schwerpunkt dieses Heftes auf Impfstrategien und Fragen der Impfkommunikation liegen. Den Autorinnen und Autoren danken wir für ihre Beiträge, den Leserinnen und Lesern wünschen wir eine anregende Lektüre.

