# Erratum zu: Impfen: Prävention von Infektionskrankheiten und ihren Folgen

**DOI:** 10.1007/s00103-025-04058-w

**Published:** 2025-05-15

**Authors:** Maren Mylius, Joseph Kuhn

**Affiliations:** 1 Abteilung 6 „Öffentliche Gesundheit“, Referat 633 „Impfungen, STIKO“, Bundesministerium für Gesundheit, Mauerstr. 29, 10117 Berlin, Deutschland; 2 Pettenkofer School of Public Health München, c/o Bayerisches Landesamt für Gesundheit und Lebensmittelsicherheit, Veterinärstr. 2, 85764 Oberschleißheim, Deutschland

**Erratum zu:**

**Bundesgesundheitsbl 2025**

10.1007/s00103-025-04033-5

Im Originalbeitrag wurden die Korrespondenzautoren unvollständig aufgeführt. Dr. Maren Mylius und Dr. Joseph Kuhn sind beide Korrespondenzautoren dieses Beitrags. Hier die vollständige Auflistung:


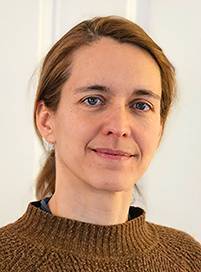


**Dr. Maren Mylius MPH**

Abteilung 6 „Öffentliche Gesundheit“, Referat 633 „Impfungen, STIKO“, Bundesministerium für Gesundheit, Mauerstr. 29, 10117 Berlin, Deutschland

Maren.Mylius@bmg.bund.de


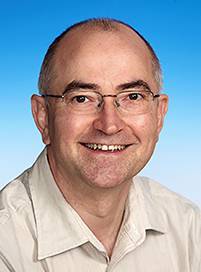


**Dr. Joseph Kuhn**

Pettenkofer School of Public Health München, c/o Bayerisches Landesamt für Gesundheit und Lebensmittelsicherheit, Veterinärstr. 2, 85764 Oberschleißheim, Deutschland

Der Originalbeitrag wurde korrigiert.

